# Wafer-level heterogeneous integration of electrochemical devices and semiconductors for a monolithic chip

**DOI:** 10.1093/nsr/nwae049

**Published:** 2024-02-26

**Authors:** Sixing Xu, Fan Xia, Zhangshanhao Li, Minghao Xu, Bingmeng Hu, Haizhao Feng, Xiaohong Wang

**Affiliations:** School of Integrated Circuits, Tsinghua University, Beijing 100084, China; College of Semiconductors (College of Integrated Circuits), Hunan University, Changsha 430001, China; School of Integrated Circuits, Tsinghua University, Beijing 100084, China; Department of Mechanical Engineering, University of California, Berkeley, CA 94720, USA; School of Integrated Circuits, Tsinghua University, Beijing 100084, China; School of Integrated Circuits, Tsinghua University, Beijing 100084, China; School of Integrated Circuits, Tsinghua University, Beijing 100084, China; School of Integrated Circuits, Tsinghua University, Beijing 100084, China; School of Integrated Circuits, Tsinghua University, Beijing 100084, China

**Keywords:** heterogeneous integration, CMOS-compatible methodology, electrochemical device, micro supercapacitor, monolithic chip

## Abstract

Micro-scale electrochemical devices, despite their wide applications and unique potential to achieve ‘More than Moore's law’, face significant limitations in constructing functional chips due to their inability to integrate with semiconductors. In this study, we propose an electrochemical gating effect and material work function matching criteria, and thus establish the first heterogeneous integration theory for electrochemical devices and semiconductors. Accordingly, we create a novel 3D integration architecture and CMOS-compatible fabrication methodology, including optimizing individual devices, electron/ionic isolation, interconnection, and encapsulation. As a demonstration, we integrate electrochemical micro supercapacitors with a P-N junction diode rectifier bridge circuit and successfully obtain the first monolithic rectifier-filter chip, which shows a revolutionary volume reduction of 98% compared to non-integrateable commercial products. The chip can provide a stable output with a tiny ripple factor of 0.23% in typical conditions, surpassing the requirements of most applications by more than one order of magnitude. More importantly, all the processes are suitable for mass production in standard foundries, allowing ubiquitous applications of electrochemistry in integrated electronics.

## Teaser

The breakthrough in heterogeneous integration theory makes it possible to integrate electrochemical devices with semiconductors as a monolithic chip, demonstrating a filter-rectifier chip that offers a remarkable 98% size reduction compared to conventional products.

## INTRODUCTION

Electrochemical devices have been widely investigated as energy storage devices, sensors, displays, and actuators, due to their high-efficiency conversion between chemical energy and electricity [1–8]. Moreover, integrating micro-scale electrochemical devices with conventional semiconductor chips holds the potential to achieve ‘More than Moore's Law’ and achieve functionalities hitherto unattainable [9], such as self-powered chips, implantable health monitoring systems, and fully integrated chemical analytical chips [10–17]. However, the substantial divergence in intrinsic mechanisms presents a formidable hurdle to achieving a monolithic electrochemical chip, referring to the heterogeneous integration of electrochemical devices with semiconductors on a single chip.

To achieve heterogeneous integration, it is imperative that the fabrication processes of electrochemical devices and semiconductors are fully compatible, ensuring no interference between the two components and guaranteeing normal chip functionality. However, crucial challenges lie in the distinct fundamental carriers in the fields of electrochemistry and semiconductors: anion/cation for electrochemistry and electron/hole for semiconductors. It results in two systems of different material, process, and operational conditions. For example, the semiconductor process requires high temperatures/pressures to achieve implantation and diffusion, which can damage vulnerable electrochemical materials [18–20]. Conversely, electrochemical materials tend to disrupt the transportation of electrons/holes, as electroactive ions easily act as extra dopants during long-term service [21,22]. Therefore, comprehensive design, including isolation and interconnection between devices, material combination, and compatible fabrication, is essential but particularly challenging to achieve heterogeneous integration [23–25].

The limitations mentioned above led to the absence of a fundamental theory of integration electrochemistry with semiconductors, let alone applicable monolithic electrochemical chips. A few experimental studies have attempted to achieve electrochemical functions on chips [26–31]. One simplified method is utilizing the conductive contact pads of integrated chips as electrodes for electrochemical sensing [28–30]. For example, Yin *et al.* deposited sensing materials on contact pads of CMOS chips, achieving a monolithic gas sensor microsystem [30]. However, this method is limited in customizing complex electrochemical devices with optimized structures, making the electrochemical application of conventional semiconductor chips more likely. Hota *et al.* demonstrated an optimized electrochemical device integrated with thin-film transistors, achieving satisfactory chip function [31]. However, this fabrication is incompatible with mainstream wafer-level silicon processes, making it impossible to utilize existing processes in foundries or achieve high-throughput batch production. Moreover, most studies are limited to the fabrication of solid parts (i.e. electrochemical electrodes), while ignoring the indispensable liquid/semi-liquid parts (i.e. electrolytes), leading to the fabricated chips being exclusive to the laboratory.

In this study, we propose an electrochemical gating effect and material work function matching criteria, and thus establish the first heterogeneous integration theory for electrochemical devices and semiconductors. Accordingly, a novel 3D integration architecture and CMOS-compatible fabrication methodology are created. We demonstrate this technique with the successful fabrication of the first monolithic rectifier-filter chip, which encompasses an electrochemical micro supercapacitor (MSC) array and P-N junction diode bridge rectifier circuit. The resulting chip has a significantly reduced volume of over 98% compared to non-integrateable commercial rectifier-filter modules, only 2.85 mm × 2.85 mm × 0.43 mm in size. After full process flow, the P-N junction diode shows a large on-off ratio of 10^4^, whilst the MSC unit displays an unprecedented capacitance density of 9.26 mF/cm^2^ (215.3 mF/cm^3^) and phase angle of −79 degrees at 120 Hz. As a result, the chip provides a stable DC output with a tiny ripple factor of 0.23% under typical conditions, surpassing the requirements of most digital chips by more than an order of magnitude. More importantly, all the processes are wafer-level and feasible for mass production in standard foundries, which will significantly accelerate the introduction of electrochemistry in integrated electronics.

## RESULTS

### Heterogeneous integration fundamental

**Figure 1.**
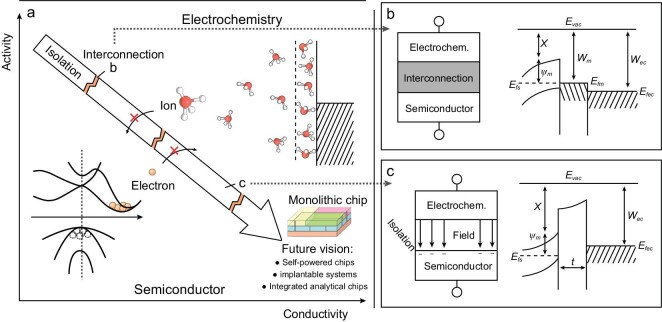
The heterogenous integration fundamental. (a) The analysis of heterogenous integration of electrochemical devices with semiconductors. (b) The structure and energy band of the interconnection part. (c) The structure and energy band of the isolation part.

The fundamental theory for heterogenous integration arises from the significant conductivity and activity differences between electrochemistry and semiconductors, as well as their mutual interference. As analyzed in Fig. 1a, electrochemical materials (electrodes/electrolytes) exhibit low conductivity and activity, in stark contrast to semiconductor materials. Therefore, semiconductor fabrication processes with high energies should be carried out first, followed by electrochemical fabrication processes, and end with the low-temperature chip encapsulation process. Meanwhile, in terms of interconnection (as depicted in Fig. 1b), the Ohmic contact between connection metal and semiconductor should be assured. This Ohmic contact requires that the work function of the connection metal does not exceed the difference between the vacuum energy level and the semiconductor Fermi energy level (for N-doped Si), as represented by Equation 1. Where ${{W}_m}$ represents the work function of the metal, ${{W}_{si}}$ represents the work function of the silicon, $\chi $ denotes the affinity energy of the semiconductor, and ${{{\mathrm{\Phi }}}_s}$ is the energy difference from the bottom of the semiconductor conduction band to the Fermi level. Moreover, the vacuum energy level difference between the connecting layer metal and the electrochemical electrode should be minimized to reduce the contact electromotive force between the two conductor interfaces. This relationship is shown in Equation 2, where ${{{\mathrm{\Phi }}}_{EC}}$ represents the vacuum energy level of the electrochemical electrode. It is challenging to find a material that satisfies all requirements considering the work functions of various metals (Table S1). Thus, a more appropriate approach is to use multi-layer connections.


(1) \begin{eqnarray*}
{{W}_m} < \chi + {{{\mathrm{\Phi }}}_s} = {{W}_{Si}} ( {for\ N - \textit{doped}\ Si} ),
\end{eqnarray*}

(2) \begin{eqnarray*}
{{{\mathrm{\Phi }}}_m} - {{{\mathrm{\Phi }}}_{EC}} \to 0.
\end{eqnarray*}

In terms of isolation, the first step is to eliminate the current leakage resulting from electron migration and ion diffusion. The interface current density (${{J}_{\mathit{inter}}}$) can be estimated approximately using Equation 3, which encompasses the Ohmic leakage current (${{J}_{ohm}}$), ion diffusion current (${{J}_{\textit{diff}}}$), and tunneling current (${{J}_{tun}}$), of which the first two contribute primarily. Since the voltage of the electrochemical device (*U*) is typically fixed, minimizing the ion concentration (${{n}_0}$) and its diffusion coefficient (*D*) becomes crucial, while simultaneously increasing the thickness (*t*) and resistivity ($\rho $) of the insulation layer.


(3) \begin{eqnarray*}
{{J}_{\textit{inter}}} = {{J}_{ohm}} + {{J}_{{diff}}} + {{J}_{tun}} \approx \frac{U}{{\rho t}} + qD\frac{{{{n}_0}}}{t}.
\end{eqnarray*}

In addition, it is imperative to prevent the electrochemical electrode/electrolyte from acting as an additional gate that alters the original operations of the semiconductors. As demonstrated in Fig. 1c, the electrochemical components operating at high voltage tend to induce an electric field on the surface layer of the semiconductors, resulting in carrier depletion or even inversion. This field effect can be assessed using Equation (4), where the electric field intensity (${{\varepsilon }_{si}}$) is approximately calculated as the ratio of the voltage applied to the electrochemical component to the insulation layer thickness multiplied by the relative dielectric constant (${{\epsilon }_r}$). It is important to ensure that this electric field intensity remains lower than the difference between the intermediate energy level (${{E}_i}$) of the semiconductor and the Fermi energy level (${{E}_f}$), divided by the inversion layer thickness (${{W}_D}$). Thus, employing high-k materials or increasing the insulation thickness can prove beneficial. Furthermore, the impact of the field effect can also be evaluated by comparing the thickness and relative dielectric constant of the insulation layer with those of the oxide layer in a standard MOSFET structure.


(4) \begin{eqnarray*}
{{\varepsilon }_{Si}} \approx \frac{U}{t} \ll \frac{{{{E}_i} - {{E}_f}}}{{q{{W}_D}}}.
\end{eqnarray*}

### 3D integration architecture and fabrication process

**Figure 2.**
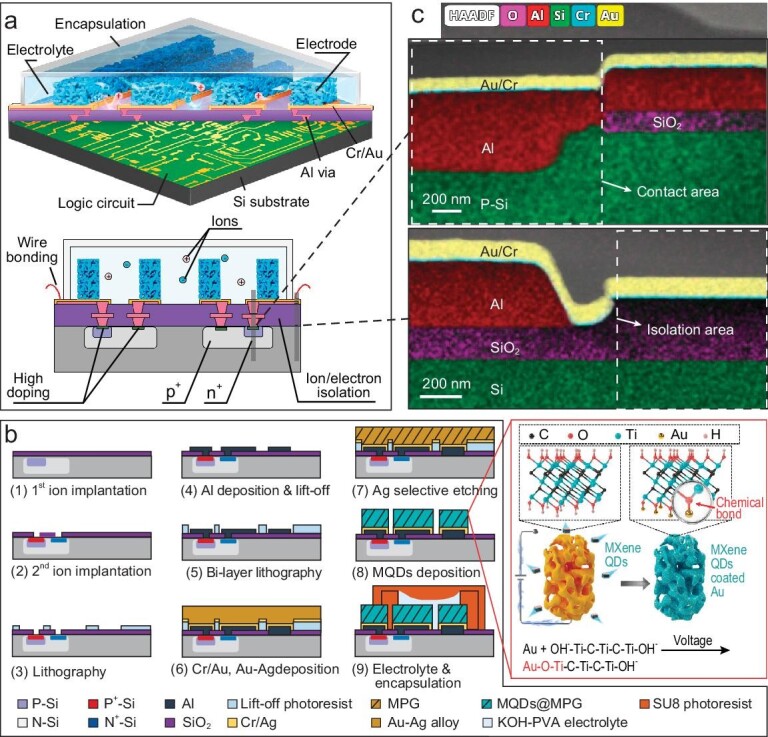
Design of the 3D integration structure and process flow of the rectifier-filter chip. (a) Full-chip and cross-section illustrations of the integrated rectifier-filter chip. (b) Main fabrication process flow of the integrated rectifier-filter chip. The right-down figure illustrates the mechanism of pseudocapacitive quantum dots decoration. (c) High-resolution TEM images of the contact area and isolation area.

As guided by the above fundamentals, we design the 3D integration architecture of a rectifier-filter chip consisting of electrochemical MSCs and semiconductor diodes, as illustrated in Fig. 2a. The chip can be divided into five parts from Si bottom to top: the semiconductor devices, isolation layer, interconnections, electrochemical electrodes/electrolytes, and encapsulation. Based on the activity difference, we first fabricate a semiconductor P-N junction diode bridge rectifier, which requires high-energy processing at the bottom. The key parameters of the diodes, including junction area, doping concentration, and implantation energy, are numerically simulated and shown in Figs S1 and S2. Above the semiconductor parts, the 350 nm thick SiO_2_ with contact holes is applied as an isolation layer to provide high electrical/ionic resistance while reducing the field effect brought by the electrochemical gates. Meanwhile, a multi-layer Al-Cr-Au structure is applied as the interconnections according to Equations 1 and 2 and the vacuum energy levels of Table S1. The Al (work function: 4.28 eV) contributes to forming Ohmic contact with deep-doped Si (calculated as 4.62 eV, Note 3), Au (5.10 eV) acts as an inert current collector for the electrochemical devices, and Cr (4.6 eV) serves as the adhesive layer in the middle. Moreover, the Al is fully covered by the Cr/Au to prevent contact with the electrochemical electrode/electrolyte, so that electrochemical side reactions can be avoided.

As for the electrochemical MSC part, we first determine the MSC unit should demonstrate a capacitance density higher than 1.08 mF/cm^2^ over 120 Hz, according to the filtering signal frequency, load impedance, and desired output ripples (Note 1). Such capacitive performance is hardly achieved by on-chip MSCs [32,33]. Therefore, we apply two unique strategies to improve the capacitive performance: (1) high-aspect-ratio on-chip framework, and (2) pseudocapacitive quantum dots decoration. The high-aspect-ratio electrode structure provides more electrochemical sites with a limited footprint; pseudocapacitive quantum dots decoration significantly improves the capacitance contribution per electrochemical site, while retaining the ion transport path and makes full use of all accessible electrode area. Those strategies effectively improve the capacitive performance of MSCs yet are more challenging to be implemented under on-chip integration circumstances. Moreover, a seamless encapsulation layer should be applied at the top to isolate the electroactive electrode and electrolyte from the ambient environment.

The detailed fabrication processes of the integrated chip are shown in Fig. 2b. First, phosphorus and boron are sequentially implanted into the silicon wafer to form P-N junction areas, followed by the plasma enhanced chemical vapor deposition (PECVD) of SiO_2_ as an isolation layer (step 1). Then, contact holes are created via dry etching of SiO_2_, and another round of ion implantations is processed to form heavy doping regions (step 2). Afterwards, the Al layer is evaporation deposited to fill the contact hole, forming an Ohmic contact with the doped silicon (step 3), followed by the deposition of the Cr/Au layer covering the Al layer (step 4). As shown in the high-resolution TEM images of Fig. 2c, a Cr/Au-Al–doped Si structure is observed in the contact area, while a Cr/Au-SiO2-Si structure is observed in the isolation area. Meanwhile, it can also be observed that the Al layer is fully covered by the Cr/Au layer, preventing its reaction with electroactive electrolytes. Next, to achieve the high-aspect-ratio electrode of the MSC, we invent a nanoporous framing technology based on a developed co-sputtering/selective etching process [34,35] and High-Aspect-Ratio Porous Electrode Lift-off (HPEL) process (steps 4–7): briefly, the bi-layer photoresist is patterned on the isolation layer, then, two kinds of metals with different electrochemical activities (in this work, Au, and Ag) are co-sputtered (step 5), and the metal with higher activity (Ag) is selectively etched to subsequently form a nanoporous structure (step 6); further, the developer passes through the nanopores of the electrodes to dissolve the bottom bi-layer photoresist, so that the unnecessary parts can be peeled off (step 7). Through this method, the thickness of the peeled-off electrode is no longer limited by the photoresist, thus high-aspect-ratio electrodes can be constructed on-chip. In addition, to implement the pseudocapacitive quantum dots decoration, we synthesize a novel two-dimensional material Ti_3_C_2_T_x_ MXene, and further prepare the MXene quantum dots (MQDs) using a hydrothermal process (see Methods section). The MQDs are only several nanometers (Fig. S3) and can diffuse into Au nanopores easily. The decoration of MQDs into the Au nanopores is achieved through a wafer-level electrochemical deposition process (step 8, see Fig. S4). The MQDs are negatively charged in the aqueous solution, hence can be deposited on the Au interface with the assistance of biased voltage, schematized in the right-down of Fig. 2b. It is important to note that the MQDs have no impact on the Au/Cr/Al contacts as they are deposited on the surface of the nanoporous Au electrode, which is positioned above the Au/Cr current collectors. As a result, the overall structure is arranged as MQDs-NPAu/Au/Cr/Al/Si, ensuring that the MQDs are completely isolated from the interfaces of Au/Cr, Cr/Al, and Al/Si. The electrodes are finally encapsulated using our developed low-temperature SU-8 packaging technique [36–38] to protect the electroactive materials from the ambient environment (step 9, see Fig. S5). As such, the wafer of rectifier-filter chips is finished.

### Characterization of the semiconductors and electrochemical devices

**Figure 3.**
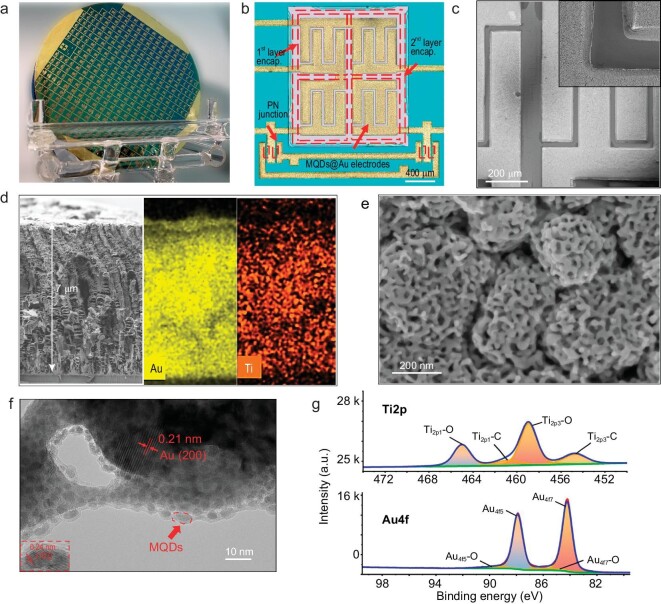
Characterization of the rectifier-filter chip. (a) Optical image of the prepared wafer. (b) Micro optical image of an integrated rectifier-filter chip with maximum working voltage of 4 V. (c) SEM characterization of the interdigital electrodes of MSC. (d) SEM characterization of the cross-section of the interdigital electrode of MSC with corresponding element maps. (e) High-resolution SEM characterization of the porous structure of the MSC electrodes. (f) HRTEM characterization of the surface structure of the MSC electrodes. (g) XPS characterization of the MSC electrodes.

The fabricated 4-inch wafer contains 332 rectifier-filter chips, as shown in Fig. 3a, of which 256 are with 4-in-series MSC array and 4 V maximum working voltage, 76 are with 2-in-series MSC array and 2 V maximum working voltage. Fig. 3b shows a detailed micro-optical image of the rectifier-filter chip with a 4-in-series MSC array. The chip volume is 2.85 mm $\times $ 2.85 mm $\times $ 0.43 mm, reduced by 98% compared to commercial circuit modules with similar functions (Note 2 and Fig. S6). The diodes are placed close to the contact pads and connected to the MSC array through Al-Cr-Au interconnections. Each unit of the MSC array is separated by the 1st layer SU-8 to prevent interference between their electric fields, and the MSC array is then encapsulated by the 2nd layer SU-8 to isolate from the environment.

The MSCs, the chip's core components, are characterized in detail. As shown in SEM images (Fig. 3c–d) of MSC electrodes, the fingers are of 150 μm width, 30 μm gap, and 7 μm thickness, separately, bringing a high aspect ratio of 0.23 for the electrode gap. The element maps of the electrode show that the Au and Ti elements are evenly distributed in the vertical direction, which indicates the uniform decoration of MQDs on the Au framework. The electrode nanoscale porous morphology is observed with specified SEM shown in Fig. 3e, in which the pore size ranges from tens to hundreds of nanometers, and is abundant for the decoration of MQDs. The interface structure of the electrode is highly concerned and investigated using high-resolution TEM (HR-TEM). In the optimized electrochemical deposition (see Methods section), the MQDs conformally decorate the nanoporous Au, as shown in Fig. 3f. As a comparison, excessive deposition of MQDs causes blockage of the nanopores, while inadequate deposition causes the incomplete coverage of the nanoporous Au, as proven in Fig. S7. The inset of Fig. 3f clearly shows a lattice space of 0.24 nm, corresponding to the (103) plane of MXene [39], which is strong evidence for the existence of MQDs. The interface structure after 1000 cycles of fully charge-discharge is characterized and shown in Fig. S8, in which the MQDs are still seamlessly decorated on the Au, proving the stability of the MQDs-Au structure. The chemical bonds of the electrode are further analyzed in the composition of the electrode. As shown in the XPS results in Fig. 3g, the Ti-C peak at 454.7 eV and Ti-O peak at 458.9 eV agrees with the peaks of MQDs in previous reports [40]. The Ti-O peak at 458.9 eV is significantly different from the 458.5 eV peak of TiO_2_ [41]. Moreover, the peak at 85.6 eV attributes to the O-Au bond at the interface of the porous Au electrode (84.2 eV), indicating the chemical-bond solid connection between MQDs and Au. The XRD and Raman characterization are not shown because they are very difficult to perform on our MQDs@ porous Au electrodes. The MQDs are small in size and conformally decorated on the porous Au for only a few layers, resulting in an extremely weak reflection signal from the MXene lattice compared to the signal from Au. Meanwhile, it is difficult to reveal the Raman shift of MXene with a large wave packet from the metal substrate. Additionally, only the MXene quantum dots located at the uppermost portion can be detected, as the porous Au electrode deflects most of the signal, leading to ambiguous results.

### Performance of the integrated chip

**Figure 4.**
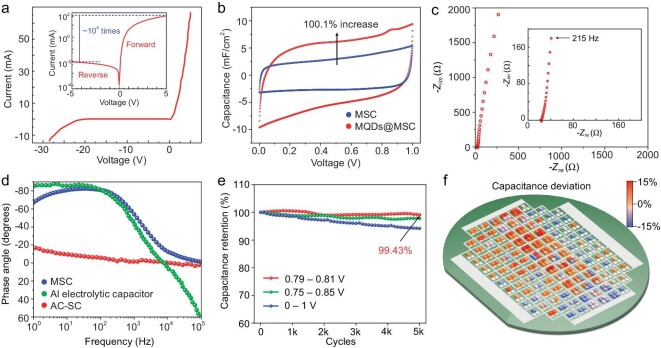
Performances of MSCs in the rectifier-filter chip. (a) Typical *I-V* curve of P-N junction diode. (b) CVs of MSCs unit with/without MQDs decoration at 10 V/s. (c) Electrochemical impedance of the MSCs. (d) Phase angle versus frequency of MSCs, commercial SC and aluminum electrolytic capacitor. (e) Cycling performances of MSCs with different operation voltage ranges (scan rate @ 10 V/s). (f) Capacitance uniformity of MSCs among the wafer.

For the testing and optimization of the individual diode and MSC unit, testing wafers are fabricated using the same techniques (Fig. S9). A typical current-voltage (*I-V*) curve of the fabricated P-N junction diode is shown in Fig. 4a. The on-off ratio is larger than 10^4^, indicating forwarding conduction and reverse cut-off. Specifically, the *I-V* curve demonstrates a forward bias voltage of 0.76 V and a backward breakdown voltage of 27 V, as shown in Fig. S10. Such results agree with the design and simulation. In addition, the full wave bridge circuit consisting of 4 diodes is also tested (Fig. S11), showing the ability of AC to DC conversion.

The performances of the MSCs are studied using electrochemical characterization methods. The cyclic voltammetry (CV) curve of the single MSC unit is plotted in red in Fig. 4b with a high scan rate of 10 V/s The curve is almost rectangular, representing a stable capacitance among the voltage window [1,2]. To study the effect of MQDs decoration, the CV curve of a similar MSC unit yet un-decorated is also plotted in blue at the same scan rate. A 100.1% capacitance improvement is obtained with the decoration, which strongly proves the effect of the pseudocapacitive quantum dots decoration strategy. Meanwhile, a slight redox peak can be observed around 0.85 V, indicating the pseudocapacitive redox reactions of MQDs. As mentioned by Yury *et al*., it is inappropriate to evaluate a microdevice based solely on its gravimetric performance. Moreover, accurately measuring the mass or effective surface area of active electrodes in on-chip devices can be exceedingly challenging. Consequently, we have opted to utilize the areal and volumetric performances for evaluating our devices. The CV result at low scan rate of 1 V/s is shown in Fig. S12 and the average capacitance densities of the MSC unit and commercial electrolytic capacitor versus scan rates are plotted in Fig. S13. The MSC unit demonstrates an average capacitance of about 9.26 mF/cm^2^ (215.3 mF/cm^3^) initially of a full device, which is more than two orders of magnitude higher than the electrolytic capacitors. The leakage current of an MSC unit with MQDs decoration is around 4 μA, as shown in Fig. S14.

The MSC frequency response is investigated via electrochemical impedance spectrometry (EIS) testing. Fig. 4c shows the typical EIS curve of an MSC unit, of which the nearly vertical orientation indicates a pure capacitive response among the testing frequency range [1,2]. The inside figure of the high-frequency region shows that the EIS curve intersects with the x-axis at around 22 Ohm, representing the device's equivalent series resistance (ESR). Considering the small area of the MSC unit, such ESR is excellent among the reported on-chip MSCs [9,10], which should be ascribed to the highly conductive electrode framework and the seamless contact between the nanoporous Au framework and Au current collector. The ESR does not directly impact the stability of the DC output which is primarily influenced by capacitance, according to Note 1. However, it does result in Joule's heat and energy loss during filtering, potentially compromising the high-frequency response of the capacitance. In future work, the aqueous electrolyte with higher ionic conductivity can be utilized to further reduce the ESR, which may rely on the development of a wafer-level CMOS-compatible liquid injection and encapsulation technology. The phase angle with frequency is an important figure of merit in evaluating the quality of a capacitor, in which the closer to −90 degrees, the better. As shown in Fig. 4d, the MSC unit demonstrates about −79.3 degrees of phase angle at the key frequency of 120 Hz, which is close to that of electrolytic capacitors. Meanwhile, the capacitance versus frequency, which is calculated according to the imaginary part of the impedance, is shown in Fig. S13. We can see that the MSC unit retains a capacitance density around 1.7 mF/cm^2^ (39.8 mF/cm^3^) at 120 Hz, and shows a time constant of 2.0 ms, meeting the design requirements. The frequency response, accompanied by the capacitive performance, strongly supports the superiority of electrochemical MSCs to conventional electrolytic capacitors in power filtering.

The life of most supercapacitors is around one million cycles [1,2], which is obviously insufficient for filtering applications. However, we prove that in an appropriate voltage window, the cycle life of MSCs can be greatly extended due to the avoidance of irreversible side reactions. As shown in Fig. 4e, when the MSC unit is fully charged with a voltage window of 1 V, the MSC remains 93.7% of original capacitance after 5000 cycles, which is consistent with the literature [9,10]. However, when controlling the voltage window between 0.75–0.85 V and 0.79–0.81 V, 97.8% and 99.4% of the capacitances can be remained at 5000 cycles, respectively. In fact, when serving as a filtering capacitor, the capacitor voltage is almost stable with the tiny ripple of several millivolts; thus, by rational design of the reference voltage, the MSC can meet the cycle life requirement. Meanwhile, to evaluate process uniformity, we measure the capacitances of all 272 MSC units among the testing wafer, as illustrated using the color grade in Fig. 4f. The maximum variances among the wafer and in an array are 12% and 3%, respectively. The variance can be further reduced by optimizing the co-sputtering technique, which contributes to around 10% of inhomogeneity among the wafer. In addition, electrochemical supercapacitors are known to experience significant current leakage, which may have notable impact in energy storage applications. However, in the context of our rectifier-filter chip, energy efficiency is not the primary concern. The primary function of this chip is signal filtering, and the current leakage does not compromise this functionality. Furthermore, this leakage is confined to the electrochemical components and does not affect the semiconductor elements or the interconnection/isolation segments. Therefore, we assert that the current leakage issue does not impede the chip's functionality.

**Figure 5.**
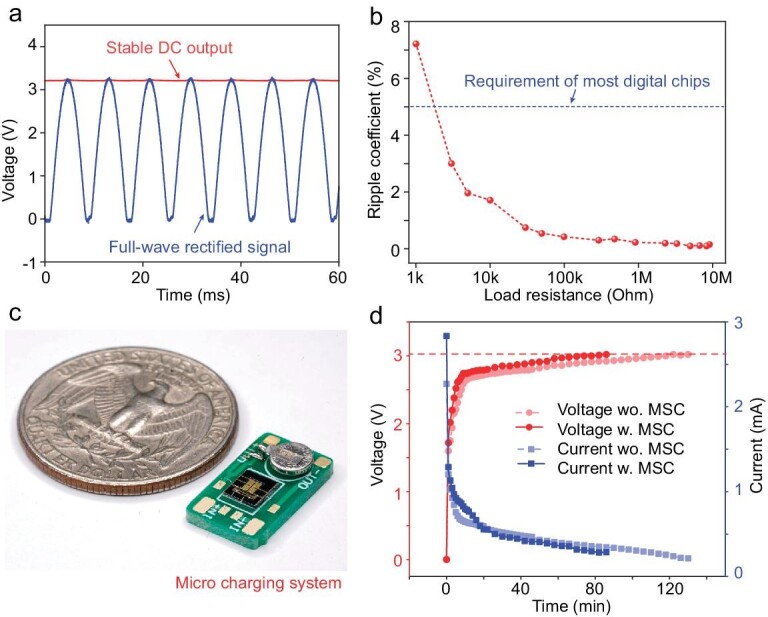
Testing of the filtering performance. (a) Output of the rectifier-filter chip under 60 Hz sinusoidal power signal with 100k Ohm load resistance. (b) Summary of ripple factors versus load resistances under 60 Hz sinusoidal power signal input. (c) Optical image of a micro charging system containing the rectifier-filter chip and a micro battery. (d) Voltage and current measurements of the micro charging system.

The rectification-filtering performance of the chips is investigated by measuring the output fluctuation under AC input with varied load resistances. Typically, a 60 Hz sinusoidal power signal is applied as input, and 100k Ohm is used as load resistance. The output signals in Fig. 5a, in which the rectified signal (blue) is symmetrical and positive, indicating the diodes are uniform and well performed. While the filtered output (red) is nearly straight, representing a DC component of 3.2 V and an AC component of 0.0073 V, which brings a tiny ripple factor of 0.23%. Moreover, the ripple factors with varied load resistances are summarized and plotted in Fig. 5b. We can see that the ripple factors decrease with the load resistance and become less than 5% when the resistance is over 3k Ohm. Considering that most digital circuits contain a negative feedback voltage regulation module in the first stage, which brings a vast input resistance over 100k Ohm [42], it is believed the MSC-based rectifier-filter chip is applicable when serving as a voltage stabilizing module in miniaturized electronics. The rectification-filtering performance is further demonstrated in a micro charging system, which contains a rectifier-filter chip and a micro battery, as pictured in Fig. 5c. The micro charging system can increase the charging efficiency and extend the battery life obviously due to the reduced input signal ripples of the battery. When subjected to a 60 Hz sinusoidal input signal, the micro charging system equipped with a 1 mAh battery can achieve a voltage of 2.8 V in just 22 min (as shown in Fig. 5d). This is a significant reduction of 52% compared to non-filtered charging, which would take 46 min. Moreover, the advantage becomes even more pronounced as the load voltage increases.

## DISCUSSION

We establish the heterogeneous integration theory for the heterogeneous integration of electrochemical devices with semiconductors, and demonstrate with the first monolithic rectifier-filter chip showing considerable superiorities in performance and size. The heterogenous integration theory, 3D integration architecture and CMOS-compatible fabrication methodology are the keys to the revolutionary performances of the individual devices and monolithic chips. More importantly, the process flow is full wafer-level, thus providing high throughput and feasibility of mass production in foundries. We believe this work proposes a general solution for the integration of various electrochemical devices with modern electronics, which may completely change the design rules of integrated circuits.

## MATERIALS AND METHODS

### Fabrication of the rectifier-filter chip

To create MQDs, we began by dissolving 1 g of LiF powder (99% purity, from Shanghai Macklin Biochemical Co., Ltd.) in 20 mL of HCl solution (9 mol/L). Then, we gradually added 1 g of Ti_3_AlC_2_ (provided by Carbon-Ukrain Co., Ltd.) to the mixture. The resulting solution was allowed to react at 40°C for 24 hours. After this period, the acidic product was washed with deionized water using centrifugation at 3500 rpm (5 min) until reaching a pH of ≥6. The washed solution was then diluted 100-fold and ultrasonicated (using equipment from Ningbo Scientz Biotechnology Co., Ltd.) for 1 hour. This mixture was centrifuged again at 4500 rpm for another hour, and the supernatant, which was believed to be a monolayer MXene solution with a concentration of ∼3.3 mg/mL, was collected. Next, the MXene solution was diluted another 100-fold and ultrasonicated for an additional 10 hours. Finally, the prepared solution was placed in a sealed reaction kettle and placed in a furnace at 120°C for 24 hours to obtain the MQD solution.

We prepared the KOH gel electrolyte by mixing KOH solution with PVA gel. Initially, 10 g polyvinyl alcohol (PVA) powder was dissolved in 90 ml of deionized (DI) water and heated to 90°C while stirring constantly to ensure clarity and transparency. Once cooled to room temperature, we added 10 ml of KOH solution (3 mol/L) to the mixture and stirred for 1 hour.

The detailed fabrication flow of silicon P-N junction diodes is illustrated in Fig. S5. First, a standard high-resistance silicon wafer was prepared and cleaned. Four times of photolithography (mask #1–4) and ion implantation were sequentially conducted, to form the n-region, p-region, highly-doped n-region, and highly-doped p-region, respectively. The concentrations and energies for those four times ion implantations were $3 \times {{10}^{12}}/{\mathrm{cm}^2}$@200k eV, $5 \times {{10}^{14}}/{\mathrm{cm}^2}$@100k eV, $4 \times {{10}^{15}}/{\mathrm{cm}^2}$@30k eV, and $4 \times {{10}^{15}}/{\mathrm{cm}^2}$@30k eV, respectively. After that, rapid thermal annealing was applied at 1050°C for 1 min, which activates the implanted ions and repairs the silicon lattice. Then, SiO_2_ with a thickness of 350 nm was grown by plasma enhanced chemical vapor deposition (PECVD) to serve as the electrical/ionic isolation layer. The SiO_2_ isolation layer was then etched to form contact holes (mask #5) by using reactive ion etching (RIE). The contact holes were then filled by the sequential deposition and pattern (mask #6) of Al layer to form an Ohmic contact with doped silicon.

To form the interdigital electrodes, a bi-layer photoresist (AR5480 for bottom, AZ601 for top) was patterned (mask #6) on the area without Al layer, followed by the sequentially sputtering deposition of Cr (10 nm) and Au (100 nm) layers, to form the multi-layer interconnection. After that, Au and Ag were co-sputtered for 100 min with the sputtering power of 500 W and 1600 W, respectively. The wafer was further immersed in 70% HNO_3_ solution for 30 min to fully remove the Ag content so that the nanoporous Au structure can be constructed. Then, the microscale high-aspect-ratio interdigital structure was built through the lift-off removal of the area with bi-layer photoresist. It should be mentioned that the thickness of the photoresist should be two times larger than that of the covered layer in conventional lift-off processes. However, in our method, the developer can reach the photoresist through the electrode nanopores so that the lift-off can be achieved even when the cover layer is much thicker than the photoresist. Then, the prepared wafer was immersed in MQDs solution for the decoration. All the electrodes were connected in this stage and acted as the working electrode in the three-electrode electrodeposition system. The decoration parameters were optimized for the 0.04 mg/mL of concentration, 1 V of applied working electrode voltage, and 10 min of deposition time. After the MSC electrode had been prepared, SU-8 2025 photoresist was patterned surrounding the MSC electrode area to serve as the 1st encapsulation layer (mask #7). The KOH gel electrolyte was then spin-coated on the wafer, followed by another pattern of SU-8 2025 photoresist (mask #8), which serves as the 2nd encapsulation layer. The two times spin-coating of SU-8 2025 photoresist were with the same spin rate of 600 rpm for 30 s followed by 3000 rpm for 60 s. Finally, the wafer was cleaned with warm water to remove the residue gel electrolyte and diced to be the proposed rectifier-filter chips.

### Characterization and measurements

X-ray photoelectron spectroscopy (XPS) surveys were performed in the binding energy range of 0–1350 eV with the X-ray photoelectron spectrometer microprobe (ESCALAB 250Xi, Thermo Fisher Scientific Inc., Waltham, Massachusetts, USA) including the specific scan data of Ti (448.5–475.5 eV) and Au (79.5–99.5 eV).

Cyclic voltammetry (CV) experiments were performed with a CHI 760E electrochemical workstation (CH Instruments, Inc., Austin, Texas, USA). The areal capacitance ${{C}_{A{\mathrm{\ }}}}$was determined from the CV response for scan rates in the range of 0.01–500 V s^–1^ using the relation:


\begin{eqnarray*}
{{C}_A} = \frac{1}{{2A\Delta V}}\displaystyle\int \frac{{IdV}}{s}.
\end{eqnarray*}

The potential range, ∆*V*, is determined by the potential, *V*, the total electrode area, *A* (including both electrode and interspace areas), the current measured during CV testing, *I*, and the scan rate, *s*.

For electrochemical impedance spectroscopy (EIS) testing, a 5 mV AC signal was applied across a frequency range of 10^0^–10^6^ Hz using an impedance/gain-phase analyzer (Solartron 1260, AMETEK Advanced Measurement Technology, Farnborough, Hampshire, UK). The real and imaginary parts of the impedance *Zʹ* and *Zʺ*, respectively, were recorded throughout this frequency range and plotted as a Nyquist plot. The capacitance *C* can be calculated using the recorded impedance data.


\begin{eqnarray*}
C = {\mathrm{\ }} - \frac{1}{{2\pi fZ^{\prime\prime}}},
\end{eqnarray*}

where *f* is the frequency.

Cycle stability tests were performed at 5000 periods in different voltage windows (0–1, 0.75–0.85, and 0.79–0.81 V) at different scan rates (100, 10, and 2 V/s) with a CHI 760E electrochemical workstation, respectively. The change of scan rates ensures the constant execution time in different voltage windows (i.e. 0.01 s). The capacitance retention is calculated every 100 periods by the areal capacitance density derived from CV curves.

Uniformity tests of the entire 118 wafer-level MSCs, including single, 2-in-series, and 4-in-series devices, were performed with a CHI 760E electrochemical workstation. The areal capacitance uniformity of every single device (i.e. four single devices in one 4-in-series MSC) was calculated using the equation:


\begin{eqnarray*}
{{{\mathrm{\Delta }}}_{\textit{single}}} = \frac{{{{C}_{A,\textit{single}}}}}{{{{C}_{\textit{mean}}}}},
\end{eqnarray*}

where ${{{\mathrm{\Delta }}}_{\textit{single}}} $ is the uniformity coefficient of every single device, ${{C}_{A,\textit{single}}}$ is the areal capacitance density of every single device, and ${{C}_{\textit{mean}}}$ is the mean value of areal capacitance of all single devices.

The demonstrations of rectifier-filter chips were made by applying a 4.6 V AC signal at 60 Hz using a waveform generator (Agilent 33220A, Agilent Technologies, Inc., Santa Clara, California, USA) where ripple factors (RFs) were determined with output waveform acquired from the signal oscilloscope (MSO-X 3034A, Keysight Technologies, Inc., Colorado Springs, Colorado, USA) using the relation:


\begin{eqnarray*}
RF = \frac{{{{V}_{AC}}}}{{{{V}_{DC}}}} = \frac{{\sqrt {{{{\left( {{{V}_{rms}}} \right)}}^2} - {{{\left( {{{V}_{DC}}} \right)}}^2}} }}{{{{V}_{DC}}}},
\end{eqnarray*}

where ${{V}_{AC}}$ is the AC voltage component of the output waveform, ${{V}_{DC}}$ is the DC voltage component which is reasonably substituted by mean values in the calculation, and ${{V}_{rms}}$ is the root-mean-square output voltage value.

## Supplementary Material

nwae049_Supplemental_File
