# Supplementary material for: Wafer-level heterogeneous integration of electrochemical devices and semiconductors for a monolithic chip
Source: Natl Sci Rev. 2024 Feb 26;11(10):nwae049. doi: 10.1093/nsr/nwae049 (PMC11409884; doi:10.1093/nsr/nwae049)
Supplement: nwae049_Supplemental_File [file nwae049_supplemental_file.pdf]

Supplementary Material

Wafer-level Heterogeneous Integration of Electrochemical Devices and Semiconductors for a Monolithic Chip

Sixing Xu[1,2#], Fan Xia[1,3#], Zhangshanhao Li[1#], Minghao Xu[1],
Bingmeng Hu[1], Haizhao Feng[1] & Xiaohong Wang[1]

[1]School of Integrated Circuits, Tsinghua University, Beijing 100084, China.

[2]College of Semiconductors (College of Integrated Circuits), Hunan University, Changsha, 430001, China.

[3]Department of Mechanical Engineering, University of California, Berkeley, CA, USA.

[#]These authors contribute equally.

Correspondence and requests for materials should be addressed to X. W. (email: wxh-ime@tsinghua.edu.cn).

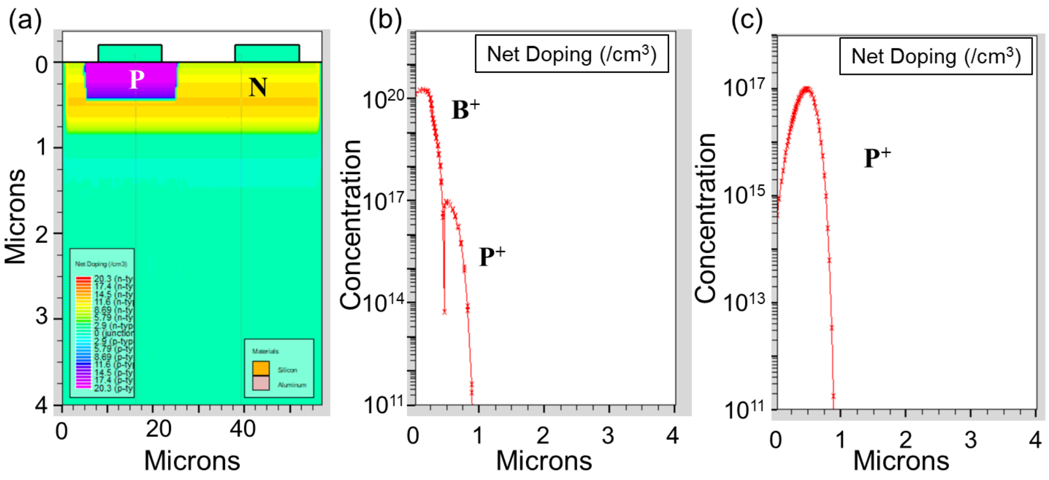

Supplementary Figure S1| Two-dimensional simulation of the doping impact to the p-n junction. (a) Cross-section simulation. (b) Concentration of the P-N region. (C) Concentration of the N-region.

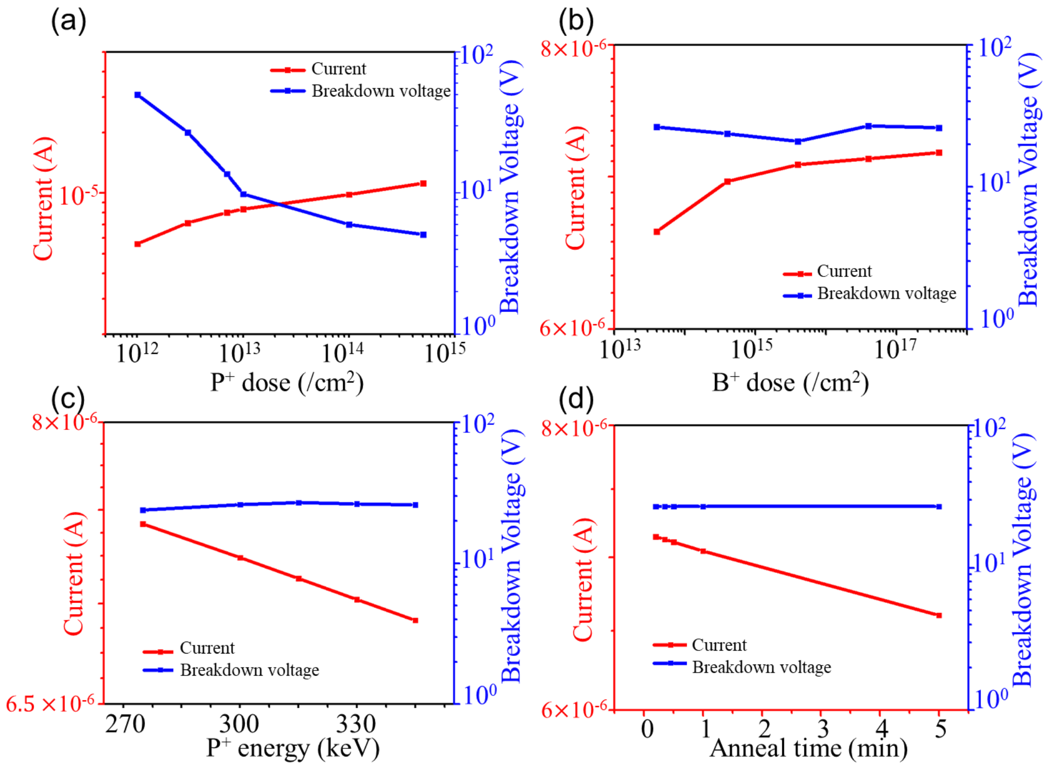

Supplementary Figure S2| Simulation of the ion implantation process. (a) Impact of P^+ dose. (b) Impact of B^+ dose. (c) Impact of P^+ energy. (d) Impact of anneal time.

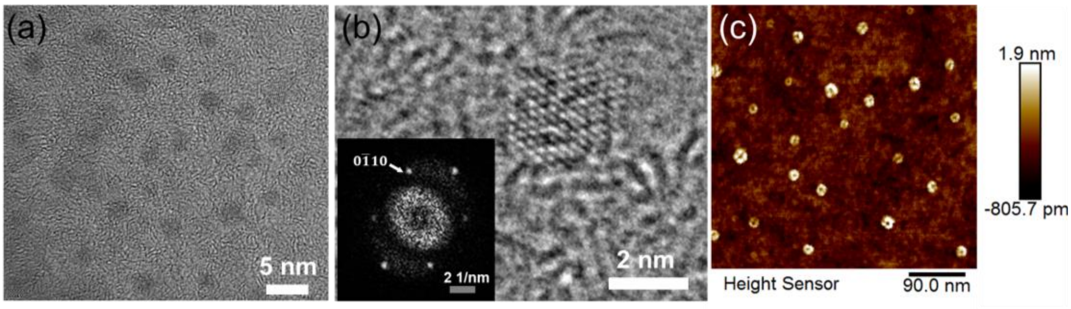

Supplementary Figure S3| Characterization of MXene quantum dots. (a)(b) TEM characterization, showing the average lateral size around 2 nm. (c) AFM characterization, proving the MXene quantum dots are monolayer.

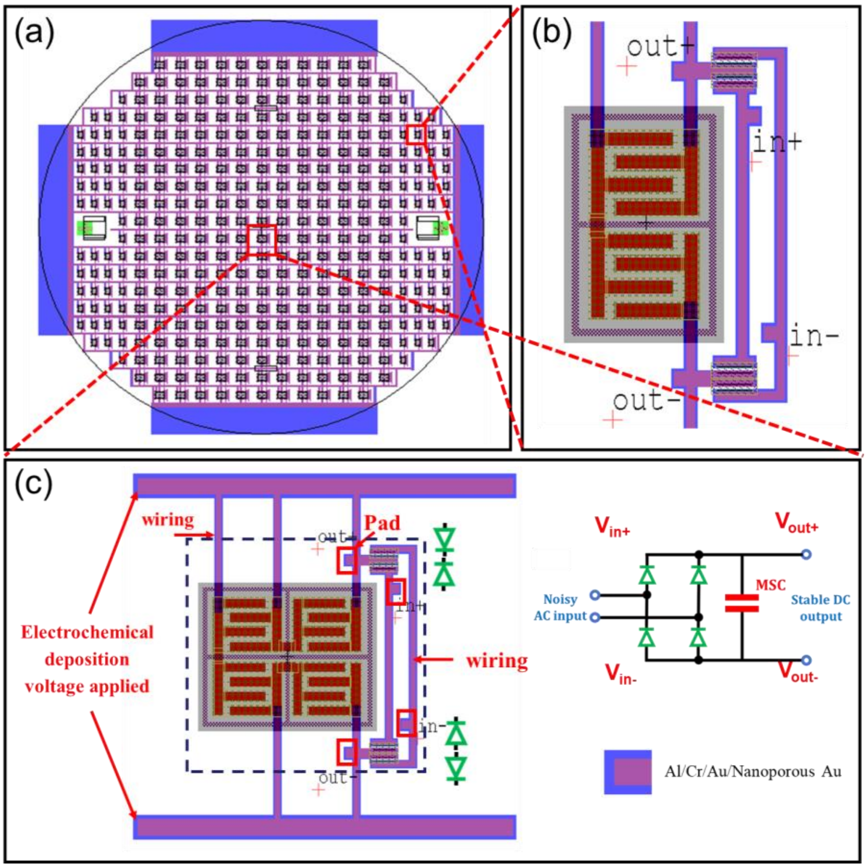

Supplementary Figure S4| Layout of the MSC-based rectifier-filter wafer. (a) Full wafer layout. (b) Illustration of the low-voltage version rectifier-filter chip. (c) Illustration of the high-voltage version rectifier-filter chip, with the equivalent circuit model.

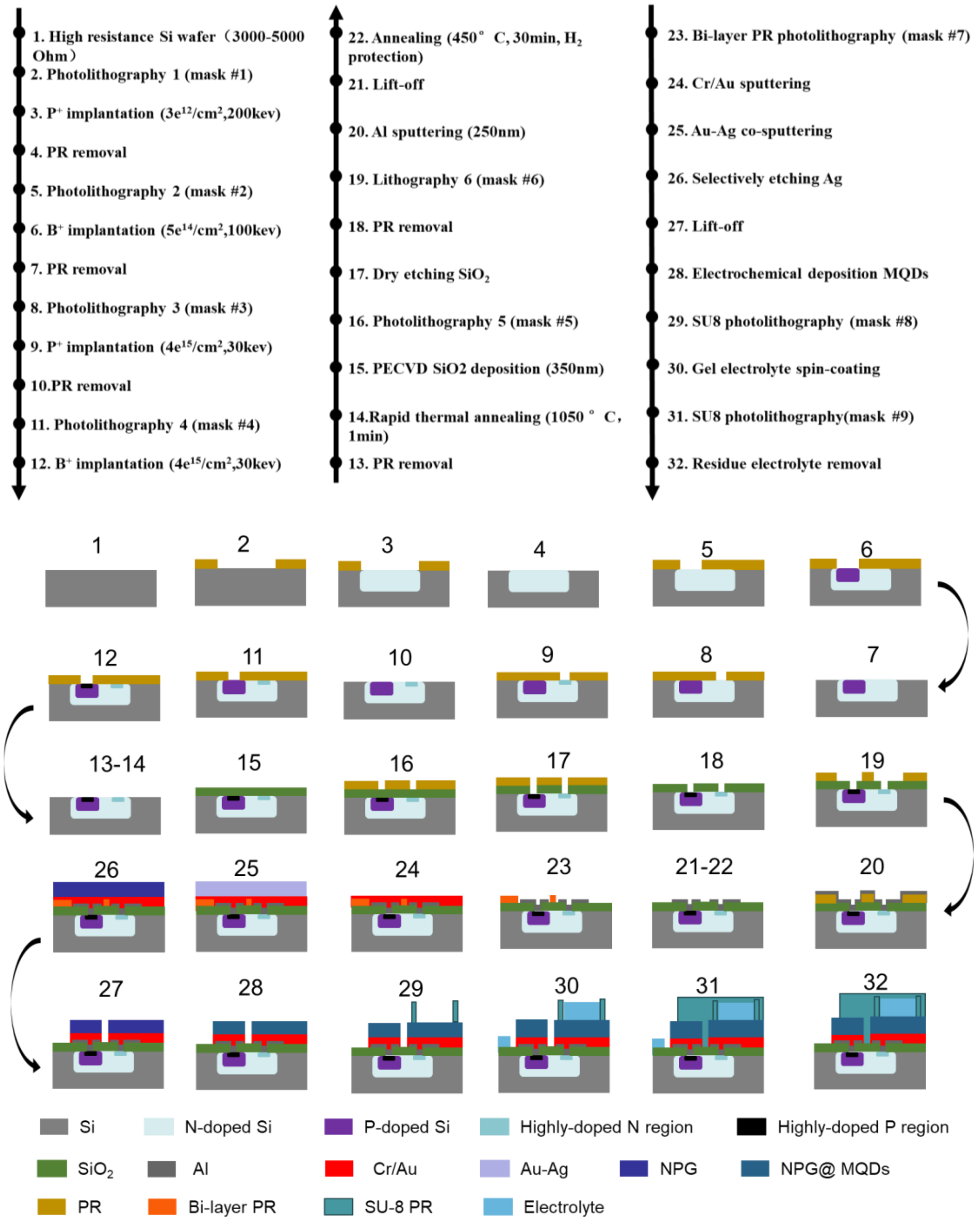

Supplementary Figure S5| Illustration of the full process of MSC-based filter-rectifier wafer.

Detailed steps:

(1) Use the high-resistance intrinsic Si wafer (3000-5000 Ω·cm) as the substrate

(2) Ultraviolet photolithography (SUSS-MA8, Photoresist AZ-P4620, 1500 rpm) with Mask #1 to define the P^+ implantation region.

(3) Ion implantation with P^+ (3×10^{12} cm^{-2} with 200 keV) to form the **N region**.

(4) Use acetone to remove the photoresist and clean the wafer.

(5) Ultraviolet photolithography (SUSS-MA8, Photoresist AZ-P4620, 1500 rpm) with Mask #2 to

define the B^+ implantation region.

(6) Ion implantation with B^+ (5×10^{14} cm^{-2} with 100 keV) to form the P region.

(7) Use acetone to remove the photoresist and clean the wafer.

(8) Ultraviolet photolithography (SUSS-MA8, Photoresist AZ-P4620, 1500 rpm) with Mask #3 to define the P^+ implantation region.

(9) Ion implantation with P^+ (4×10^{15} cm^{-2} with 30 keV) to form the **heavily doped N^+ region** (**Ohmic Contact**).

(10) Use acetone to remove the photoresist and clean the wafer.

(11) Ultraviolet photolithography (SUSS-MA8, Photoresist AZ-P4620, 1500 rpm) with Mask #4 to define the B^+ implantation region.

(12) Ion implantation with B^+ (4×10^{15} cm^{-2} with 30 keV) to form the **heavily doped P^+ region** (**Ohmic Contact**).

(13) Use acetone to remove the photoresist and clean the wafer.

(14) Rapid thermal annealing (1050 °C with 1 minute) to activate the P^+ and B^+ ions.

(15) Plasma Enhanced Chemical Vapor Deposition (PECVD) to form the SiO_2 insulating layer (350 nm).

(16) Ultraviolet photolithography (SUSS-MA8, Photoresist AZ-P4620, 1500 rpm) with Mask #5 to define the ohmic contact region.

(17) Dry etching the SiO_2 to form the vias of ohmic contacts in P^+ and B^+ regions.

(18) Use acetone to remove the photoresist and clean the wafer.

(19) Ultraviolet photolithography (SUSS-MA8, Photoresist AZ-601, 3000 rpm) with Mask #6 to define the Al contact regions.

(20) Magnetron sputtering Al (DC 200W, 250 nm).

(21) Use acetone to lift off the Al metal to form the ohmic contact.

(22) 30-minute annealing under 450 °C with the protection from H_2.

(23) Ultraviolet photolithography with the bi-layer photoresist (SUSS-MA8, Photoresist AZ-5480, 2000 rpm, Photoresist AZ-601, 3000 rpm) with Mask #7 to define the MSC region.

(24) Magnetron sputtering Cr (DC 1600W, 10 nm)/Au (RF 350W, 100 nm).

(25) Magnetron co-sputtering Au (RF 350W, 60 minutes) and Ag (DC 1000W, 60 minutes).

(26) Use the HNO_3 (70 wt.%, 30 minutes) to selectively remove Ag from Au/Ag alloy, forming the porous Au.

(27) Use acetone to lift off the Cr/Au/porous Au to form the framework of MSCs.

(28) Electrochemical deposition (Three-electrode system, Voltage 1 V, 10 minutes) MXene quantum dots to conformally incorporate with porous Au.

(29) Ultraviolet photolithography with the negative photoresist (SUSS-MA8, Photoresist SU8-2025, 2000 rpm) with Mask #8 to define the encapsulation region.

(30) Spin-coat the gel electrolyte (3M KOH + PVA, 3500 rpm) onto the wafer.

(31) Ultraviolet photolithography with the negative photoresist (SUSS-MA8, Photoresist SU8-2025, 1500 rpm) with Mask #9 to finish the encapsulation.

(32) Use the warm water (50 °C, 20 minutes) to remove the residual gel electrolyte on other regions of wafer.

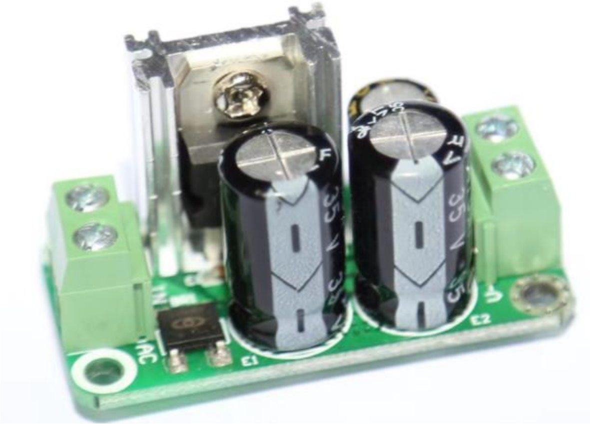

Supplementary Figure S6| Conventional un-integratable product for rectifier filter. Source: https://ic-item.jd.com/10038723417555.html#crumb-wrap. Estimation of the size reduction: considering the required electrolytic capacitor (4.7 μF) and PCB board, the minimum size should be $5 \times 5 \times 7\ \text{mm}^3$.

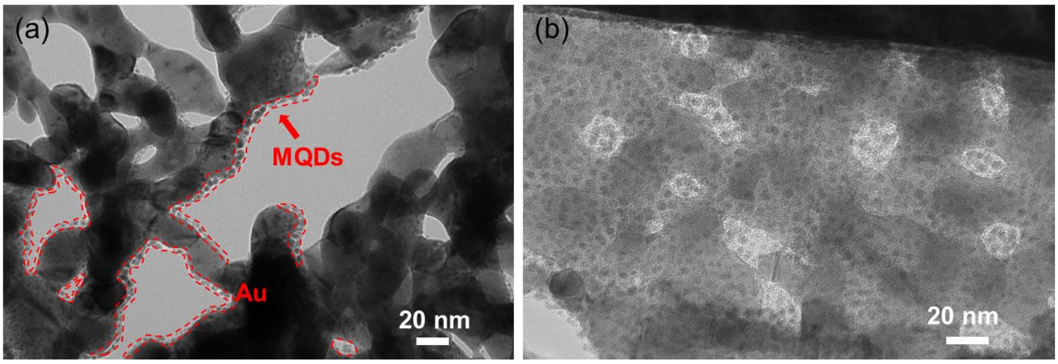

Supplementary Figure S7| TEM characterizations of the MXene quantum dots decoration onto Au nanopores. (a) Nanopores with too few and (b) too much MXene quantum dots.

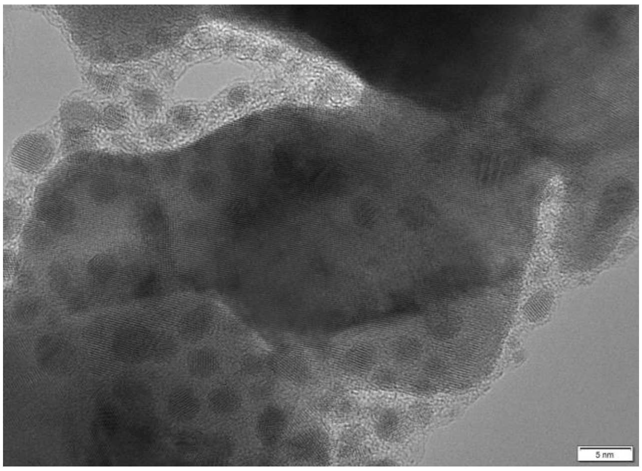

Supplementary Figure S8| TEM characterizations of the MXene quantum dots decoration onto Au nanopores after 1000 cycles charge-discharge.

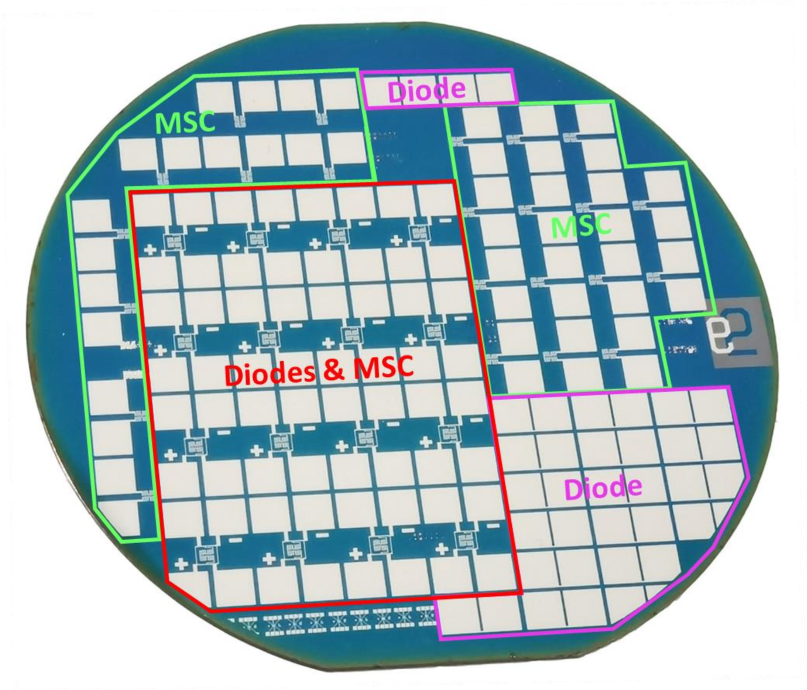

Supplementary Figure S9| Photograph of the testing wafer.

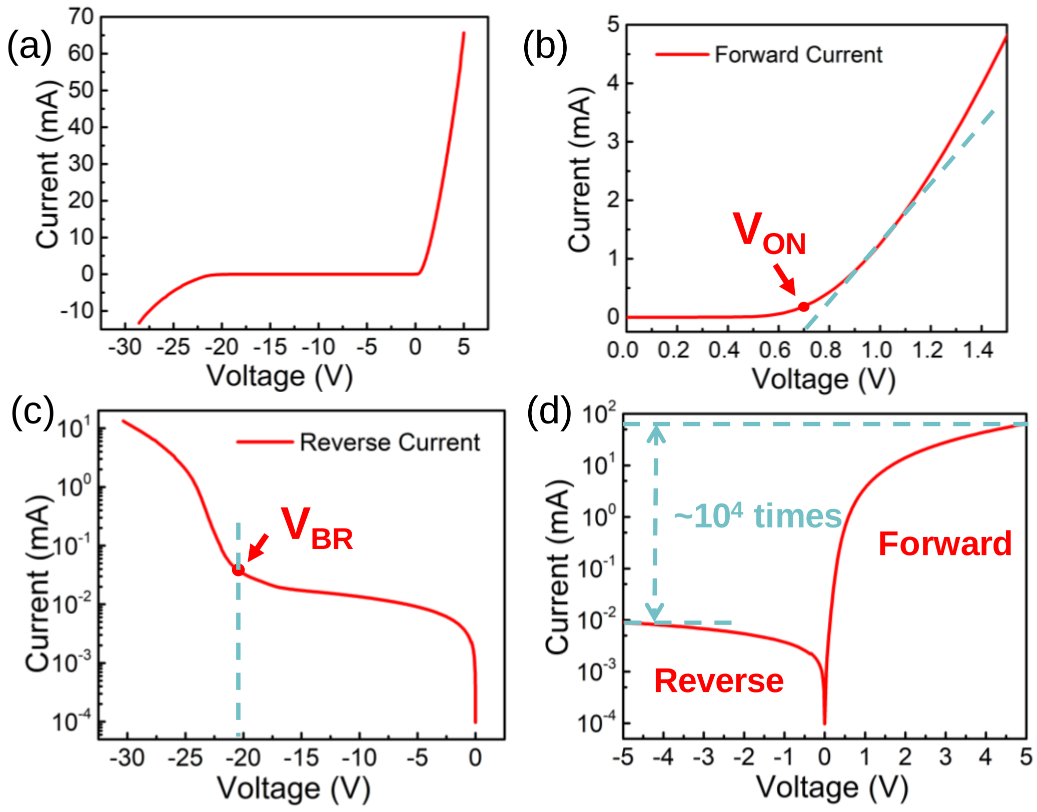

Supplementary Figure S10| IV curves of the fabricated PN junction.

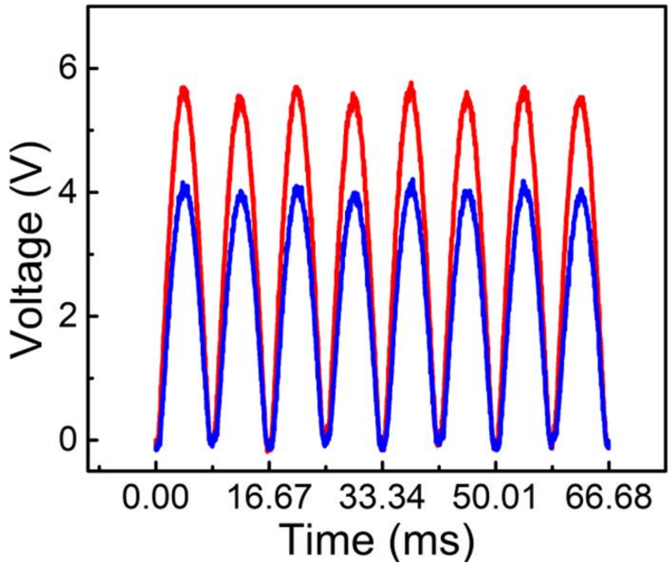

Supplementary Figure S11| The rectified outputs under different AC inputs (input voltage amplitude for red curve: 8V, input voltage amplitude for blue curve: 6V)

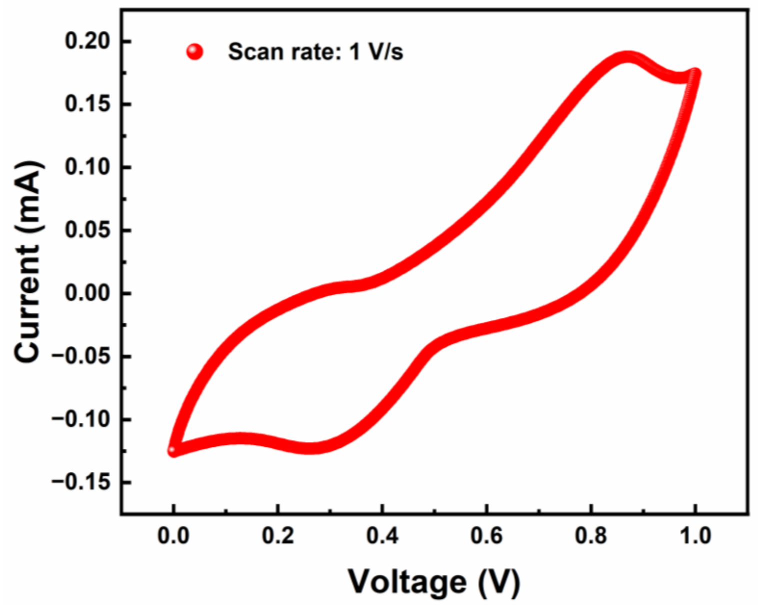

Supplementary Figure S12| The CV of MSC with MQDs decoration at 1 V/s.

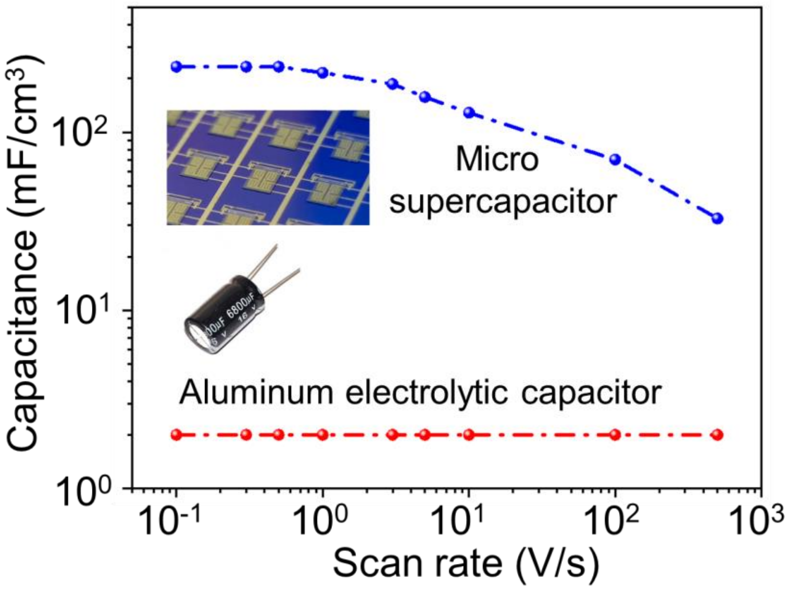

Supplementary Figure S13| The average capacitance densities of MSC unit and commercial electrolytic capacitor versus scan rates.

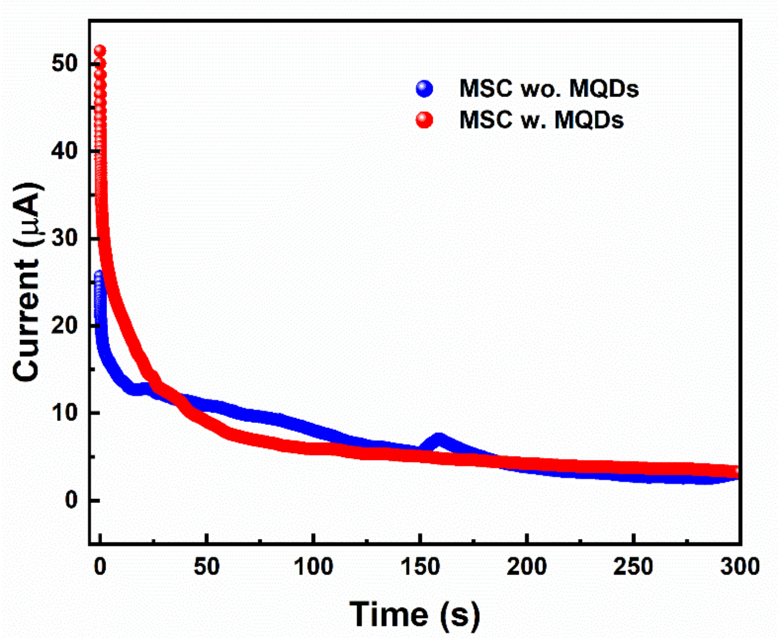

Supplementary Figure S14| The leakage current of MSCs with/without MQDs. Method: maintaining the voltages of MSCs at 1V and measuring the currents.

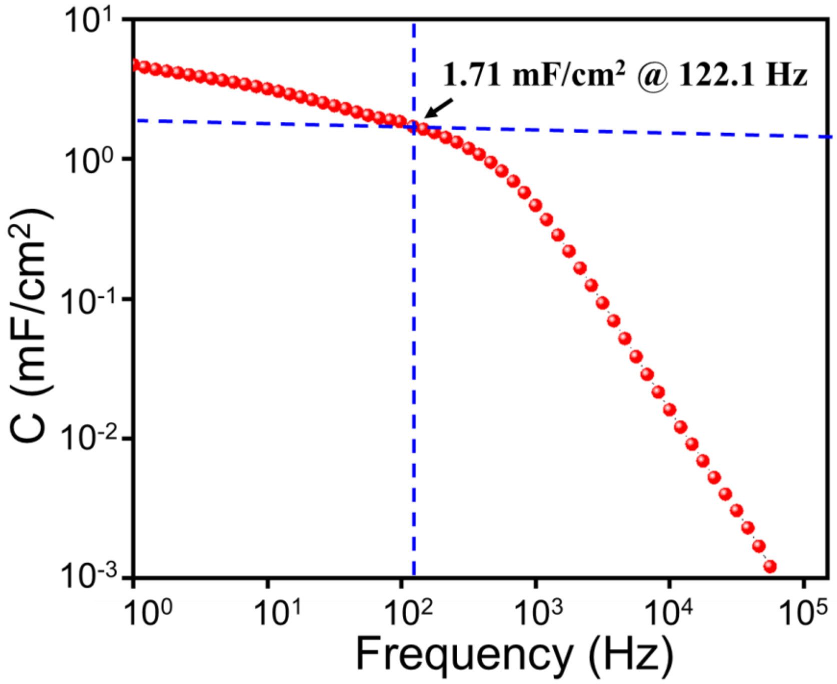

Supplementary Figure S15| The capacitance density of MSC unit versus frequency.

Supplementary Table S1| Work functions of various materials.

**Element**	**Work function**	**Element**	**Work function**
Ag	4.26-4.74	Sn	4.42
**Au**	**5.1-5.47**	Tb	3.00
Be	4.98	Ti	4.33
Ca	2.87	V	4.3
Co	5	Yb	2.60
Cu	4.53-5.1	**Al**	**4.06 – 4.26**
Ga	4.32	B	~4.45
Hg	4.475	Bi	4.31
K	2.29	Cd	4.08
Lu	~3.3	**Cr**	**4.5**
Mo	4.36-4.95	Eu	2.5
Nd	3.2	Gd	2.90
Pb	4.25	In	4.09
Rb	2.26	La	3.5
Ru	4.71	Mg	3.66
Se	5.9	Na	2.36
Ni	5.04 – 5.35	Fe:	4.67 – 4.81
Pd	5.22 – 5.6	Hf	3.9
Re	4.72	Ir	5.00 – 5.67
Sb	4.55 – 4.7	Li	2.9
**Si**	**4.60 – 4.85**	Mn	4.1
Sr	~2.59	Nb	3.95 – 4.87
Te	4.95	Os	5.93
Tl	~3.84	Pt	5.12 – 5.93
W	4.32 – 5.22	Rh	4.98
Zn	3.63 – 4.9	Sc	3.5
As	3.75	Sm	2.7
Ba	2.52 – 2.7	Ta	4.00 – 4.80
C	~5	Th	3.4
Ce	2.9	U	3.63 – 3.90
Cs	2.1	Y	3.1
**SiO_2**	**5**	Zr	4.05

Ref[1]: Lide, D.R. ed., 2004. CRC handbook of chemistry and physics (Vol. 85). CRC press.

Supplementary Table S2| A comparison of our device with the most represented reported devices in labs and commercial markets.

	**This work**	**Commercial capacitor***	**Ref [1]**	**Ref[2]**	**Ref[3]**	**Ref[4]**	**Ref[5]**
**Areal Cap. Density (DC) (mF/cm^2)**	9.26	0.29	8	0.31	0.3	N/A	1.3
**Areal Cap. Density (120 Hz) (mF/cm^2)**	1.21	0.29	6	0.23	0.15	0.08	0.5
**Volume Cap. Density (DC) (mF/cm^2)**	215.3	0.56	226	7.75	7.5	N/A	N/A
**Volume Cap. Density (120 Hz) (mF/cm^2)**	28.1	0.56	171	5.75	3.75	6.4	N/A
**Leakage Current** (μA)	4	3	N/A	N/A	N/A	N/A	N/A
**Phase Angle (120 Hz) ^{o}**	-79	-80	-80	-81.5	-80.5	-82.6	-73
**On-chip**	Yes	No	No	Yes	Yes	No	Yes
**Semiconductor integrability**	Yes	No	No	No	No	No	No

**: From datasheet of commercial electrolytic capacitor EMVA4R0ARA470MD55G (https://www.chemi-con.co.jp/products/relatedfiles/capacitor/catalog/MVARA-e.PDF)*

Ref[1]: Xu, S. et al. Nano Energy 58, 803-810 (2019).

Ref[2]: Lin, J. et al. Nano Lett. 13, 72-78 (2013).

Ref[3]: Kurra, N. et al. ACS Appl. Mater. Interfaces 8, 12748-12755 (2016)

Ref[4]: Yuan, Y. et al. Sci. China Mater. 65, 2412-2420 (2022)

Ref[5]: Yang, C. et al. Angewandte Chemie International Edition 56, 14, pp.3920-3924 (2019)

Some performances are missing in the literatures; the calculation of volume performances include the volume of the substrate and encapsulation.

Supplementary Note 1: Estimation of the MSC capacitance required for filtering

The basic circuit diagram and signal waves of the rectifier-filter module are illustrated in Fig. S9. Assuming the output has reached a steady state, so that the signal rise and fall in each cycle are equal. During the discharge period, the voltage drops exponentially with the time constant of R_LC. Actually, such time constant is much larger than the signal period, and the discharge time is much longer than the charge time. Therefore, the following approximation can be made:

$$V_{drop} = V_0 \times (1 - e^{-\frac{\Delta t}{R_L C}}) \approx V_0 \times (1 - e^{-\frac{T}{2R_L C}}) \approx \frac{V_0 T}{2R_L C} \qquad (1)$$

V_{drop} is the voltage drop during each discharge process, V_0 is the maximum voltage, Δt is the discharge time, C is the capacitance, R_L is the input resistance of the next stage circuit, T is the period of the input signal (usually 1/60 second). Meanwhile, the average voltage of the output signal can be approximately calculated:

$$V_{ave} \approx V_0 - \frac{1}{2}V_{drop} \approx V_0(1 - \frac{T}{4R_L C}) \qquad (2)$$

V_{ave} is the average voltage of the output. In such, the ripple factor of the output can be calculated:

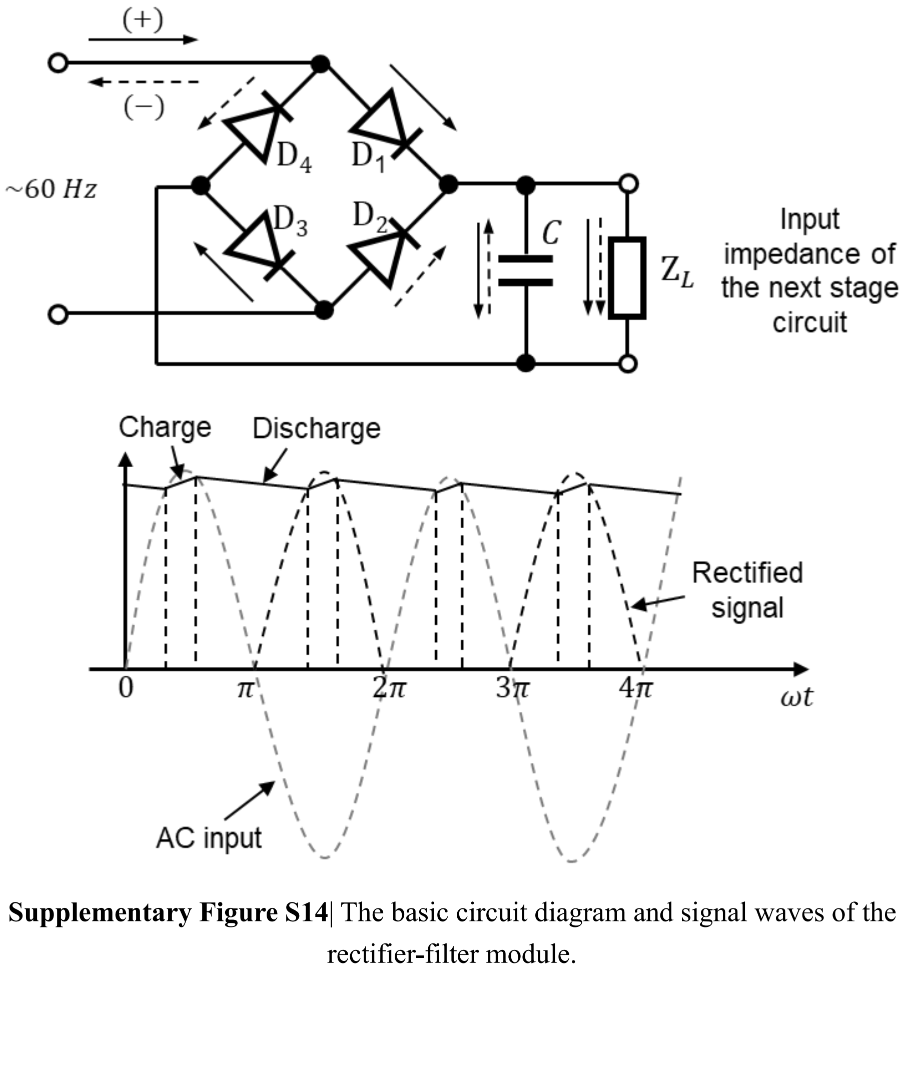

Supplementary Figure S14| The basic circuit diagram and signal waves of the rectifier-filter module.

$$n_{ripp} = \frac{V_{ac}}{V_{dc}} \approx \frac{V_{drop}}{2V_{ave}} \approx \frac{1}{\frac{4R_L C}{T} - 1} \tag{3}$$

In typical digital circuit applications, the rectifier-filter module is connected to a negative feedback voltage regulation module. The input resistance of the negative feedback voltage regulation module is usually equal to the input resistance of a common-gate transistor, which is about 100k Ohm. To reach the required 1% of ripple factor, it can be calculated as:

$$n_{ripp} \approx \frac{1}{\frac{4R_L C}{T} - 1} < 1\% \tag{4}$$

$$C > \frac{101T}{4R_L} = 4.2 \times 10^{-3} (mF) \tag{5}$$

Assuming the area for the MSC is about the area of a chip, like 2.5mm × 2.5mm. Meanwhile, it should be mentioned that MSCs should be 4-in-series connection to meet the voltage required, which means the effective capacitance density should be divided by 16. Hence, the required capacitance density of the MSC unit should be:

$$C_{req} = \frac{4.2 \times 10^{-3}}{6.25} \times 16 \times 100 \approx 1.08 (mF / cm^2) \tag{6}$$

Supplementary Note 2: Calculation of the volume reduction

The size of the fabricated rectifier-filter chip is 2.85 mm × 2.85 mm × 0.43 mm. In the market, the minimum size of electrolytic capacitor with similar capacitance (4.7 μF) should be 4 mm for diameter and 5.4 mm for the height. Considering the spaces for the discrete diode, contact pads and PCB board, the reasonable size for the commercial product should be $5mm \times 5mm \times 7mm$. Therefore, the volume reduction can be calculated to be around 98%.

Supplementary Note 3: Calculation of the work function

Take the N^+ region as the example, and suppose all dopants are completely ionized at room temperature, then we have:

$$n_0 = N_D = N_C \exp\left(-\frac{E_C - E_F}{kT}\right)$$

thus,

$$E_F = E_C + kT\ln\left(\frac{N_D}{N_C}\right) = E_C + 0.026\ln\left(\frac{4 \times 10^{15}}{2.8 \times 10^{19}}\right) = E_C - 0.23\ eV$$

since,

$$\Phi_s = E_C - E_F = 0.23\ eV$$

which means,

$$W_{Si} = \chi + \Phi_s = 4.39 + 0.23 = 4.62\ eV$$

hence,

$$\Phi_m(4.28\ eV) < W_{Si}(4.62\ eV)$$
